# The Electrosome: A Surface-Displayed Enzymatic Cascade in a Biofuel Cell’s Anode and a High-Density Surface-Displayed Biocathodic Enzyme

**DOI:** 10.3390/nano7070153

**Published:** 2017-06-23

**Authors:** Alon Szczupak, Dror Aizik, Sarah Moraïs, Yael Vazana, Yoav Barak, Edward A. Bayer, Lital Alfonta

**Affiliations:** 1 Department of Life Sciences and the Ilse Katz Institute for Nanoscale Science and Technology, P.O. Box 653, 8410501 Beer-Sheva, Israel; alonszcz@post.bgu.ac.il (A.S.); dror12321@gmail.com (D.A.); 2 Department of Biomolecular Sciences, Weizmann Institute of Science, 234 Herzl St., P.O. Box 26, 7610001 Rehovot, Israel; sarahv@weizmann.ac.il (S.M.); yael.vazana@sial.com (Y.V.); ed.bayer@weizmann.ac.il (E.A.B.); 3 Department of Chemical Research Support, Weizmann Institute of Science, 234 Herzl St., P.O. Box 26, 7610001 Rehovot, Israel; yoav.barak@weizmann.ac.il

**Keywords:** hybrid biofuel cells, enzymatic cascades, scaffoldin, cohesin, dockerin, redox enzymes, cellulosome

## Abstract

The limitation of surface-display systems in biofuel cells to a single redox enzyme is a major drawback of hybrid biofuel cells, resulting in a low copy-number of enzymes per yeast cell and a limitation in displaying enzymatic cascades. Here we present the electrosome, a novel surface-display system based on the specific interaction between the cellulosomal scaffoldin protein and a cascade of redox enzymes that allows multiple electron-release by fuel oxidation. The electrosome is composed of two compartments: (i) a hybrid anode, which consists of dockerin-containing enzymes attached specifically to cohesin sites in the scaffoldin to assemble an ethanol oxidation cascade, and (ii) a hybrid cathode, which consists of a dockerin-containing oxygen-reducing enzyme attached in multiple copies to the cohesin-bearing scaffoldin. Each of the two compartments was designed, displayed, and tested separately. The new hybrid cell compartments displayed enhanced performance over traditional biofuel cells; in the anode, the cascade of ethanol oxidation demonstrated higher performance than a cell with just a single enzyme. In the cathode, a higher copy number per yeast cell of the oxygen-reducing enzyme copper oxidase has reduced the effect of competitive inhibition resulting from yeast oxygen consumption. This work paves the way for the assembly of more complex cascades using different enzymes and larger scaffoldins to further improve the performance of hybrid cells.

## 1. Introduction

Biofuel cells are electrochemical devices that use enzymatic reactions to catalyze the conversion of chemical energy to electricity in a fuel cell. They can be classified as microbial fuel cells (MFCs), which use living microorganisms [1,2], or enzymatic fuel cells, which use purified enzymes [3,4]. Hybrid biofuel cells combine the characteristics of both classes of biofuel cells. The concept was initially introduced by the use of redox enzymes surface-displayed on different microorganisms and in biofuel cells [5,6]. In the first study, glucose oxidase was displayed on the surface of *Saccharomyces cerevisiae* (*S. cerevisiae*) yeast using the a-agglutinin yeast surface-display (YSD) system [7] for use in the anode of a biofuel cell with an enzymatic based laccase cathode. The performance of the cell was higher than those employing either an enzymatic glucose oxidase anode or a microbial *S. cerevisiae*-based anode and similar to that utilizing an anode consisting of both the free enzyme and yeast. The phenomenon has been termed ‘the additive effect’. In a later study, the oxygen-reducing enzymes laccase and bilirubin oxidase were coupled to the anode, and a fully hybrid biofuel cell was developed [8]. Yet another advantage of enzyme surface-display is that the laborious and time-consuming purification processes of enzymes were avoided and the surface-displayed redox enzymes were site-specifically wired to an electrode to further improve electron transfer, as described by Amir et al. [9], who used the *Escherichia coli* (*E. coli*) autodisplay system [10] for the display of alcohol dehydrogenase (ADH) as the anodic reaction catalyst. This system was further improved to assemble an artificial biofilm of bacteria surface-displaying ADH [11]. In situ processing of complexed polysaccharides such as starch into fuel was demonstrated in a hybrid cell by the coupling of yeast displaying the starch-hydrolyzing enzyme glucoamylase with glucose oxidase-displaying yeast in a mixed culture [12]. A mixed culture, however, cannot be efficiently controlled over time. In this context, hybrid biofuel cells of yeast displaying different hydrogenases on the same surface were presented [13].

In the microbial fuel cells, the metabolic pathways of the organism provide full cascades of redox enzymes, which can catalyze the full oxidation of various fuels. The microorganisms grow and divide, and that keeps the system alive and able to generate more catalyst for long-term usage. However, such systems suffer from poor electron transfer to the electrode and low coulombic efficiency due to multiple losses to the environment and to competing processes in the microorganisms. This results in a less-defined system, which renders cell potential harder to control over time. In addition, mass transfer problems, owing to the need for the fuel to cross the cellular membrane, result in a loss of power outputs.

The use of enzymatic cascades in enzymatic fuel cell anodes resulted in very high power outputs, as the electron density achieved was much higher when the fuel was fully oxidized; thus all electrons extracted from a fuel molecule could be transferred to the anode [14,15,16,17]. The different surface-display systems presented in different microorganisms and employed in hybrid fuel cells use the fusion of the surface-displayed enzyme to a surface-displayed protein [5,8,9,12]. One of the major drawbacks of the hybrid systems is the limitation of only one copy of an enzyme to be surface-displayed per each surface-displayed protein, significantly limiting the number of redox enzymes per yeast cell. This also limits the ability to display an enzymatic cascade, as each enzyme needs to be displayed using a different vector, which adds complexity to the system. As the number of surface proteins in the cell is limited, the insertion of more than one YSD vector, each encoding for another enzyme, will result in competition over a limited number of sites and an overall loss in the cascade units. A second limitation of the hybrid cell is that the displayed redox enzyme has to be expressed by the host organism carrying the surface-display system, which is not ideal for enzyme overexpression and may result in lower enzymatic activity.

These two challenges require a substrate-channeling approach in order to efficiently exploit most of the electrons of each fuel molecule. Substrate-channeling methods involve the design of systems in which several catalysts are designed to act in proximity, which can significantly improve the total efficiency of a cascade of reactions [18]. Different examples for such channeling methods were reported before, where different designs and approaches were used. Van Nguyen et al. used a DNA scaffold in order to catalyze a cascade of reactions in which invertase of *S. cerevisiae* was coupled to *Aspergillus niger*’s glucose oxidase to present a cascade in which sucrose was hydrolyzed by the former enzyme, followed by an oxidation by the latter [19]. A native substrate-channeling system is inherent in the bacterial cellulosome, in which multiple cellulolytic enzymes are bound to a non-catalytic protein, called scaffoldin, which is displayed on the bacterial cell surface [20,21]. The scaffoldin protein consists of several modules named cohesins, which bind with high affinity to the complementary dockerin modules borne by the cellulosomal enzymes [22]. The binding of the scaffoldin to the bacterial cell occurs via a second type of dockerin module in the scaffoldin protein, which binds to a cohesin module of an anchoring scaffoldin bound to the cell surface. In a defined bacterial species, different cellulose-degrading enzymes with complementary activities share similar dockerin modules of like specificities, and the enzymes are thus bound randomly to the scaffoldin protein [23,24], generating heterogeneous cellulosomes. However, since the cohesin-dockerin interaction is species specific [25], scaffoldin chimeras could also be generated by fusing genes encoding for cohesins from different microorganisms via short protein linkers, thus generating designer cellulosomes [26,27,28,29]. In designer cellulosomes, the number of copies and the location of any enzyme in the scaffoldin can be controlled thus an enzymatic cascade can be self-assembled [30,31].

The gene encoding for the chimeric scaffoldin can be surface-displayed in microorganisms, thus enabling the attachment of multiple enzymes [32,33,34]. Here we designed a system in which the dockerin-containing redox enzyme will be overexpressed in *E. coli* and the lysate will be incubated with the yeast displaying a chimeric scaffoldin on their surface, thereby avoiding the need to purify the redox enzymes. Such a cascade for the oxidation of methanol has been presented before [35]. However, electrochemical activity and/or performance in a biofuel cell was not demonstrated. Here, we have employed cellulosome machinery and terminology and generated a redox enzymatic cascade to form what we have termed: an electrosome. The advantage in such a system is that we exploit the versatility and robustness of the YSD system in yeast while expressing redox enzymes in *E. coli*, thus avoiding post-translational modifications (e.g., glycosylation), that occur in yeast, which would interfere with the electron-transfer processes to and from the electrode.

Unlike the additive effect in the anode compartment observed in our earlier studies [5], in the cathode compartment, a decrease in the hybrid biofuel cell’s performance was observed due to competition over oxygen between the surface-displayed oxygen-reducing enzyme and the aerobic respiration of yeast [8]. Yet, when the yeast cells in the hybrid biofuel cell were subjected to conditions of anaerobic respiration, the advantage of the hybrid biofuel cell during long-term operation was evident since the performance of the enzymatic cell decreased with time. Yeast anaerobic respiration can be achieved by the addition of antimycin A, which inhibits yeast oxygen respiration [36]. Adding antibiotics is not a sustainable long-term solution since it is expensive and requires the continuous addition of temperature-sensitive antibiotics to the fuel cells. These considerations are even more relevant when using the biofuel cell in a continuous flow cell mode. An alternative approach to overcome competitive inhibition is to increase the enzyme copy number or the density of surface-displayed enzymes while keeping the yeast cell growth phase in a steady state. The approach of scaffoldin surface-display enables an increase of the density of the cathodic biocatalyst on the yeast surface by virtue of a scaffoldin protein that contains several cohesin domains displayed using the YSD system, to which multiple copies of a single type of a dockerin-containing oxygen-reducing enzyme are bound. In such a system, a monovalent scaffoldin may be used as all the enzymes are identical and binding specificity is not required.

**Figure 1:**
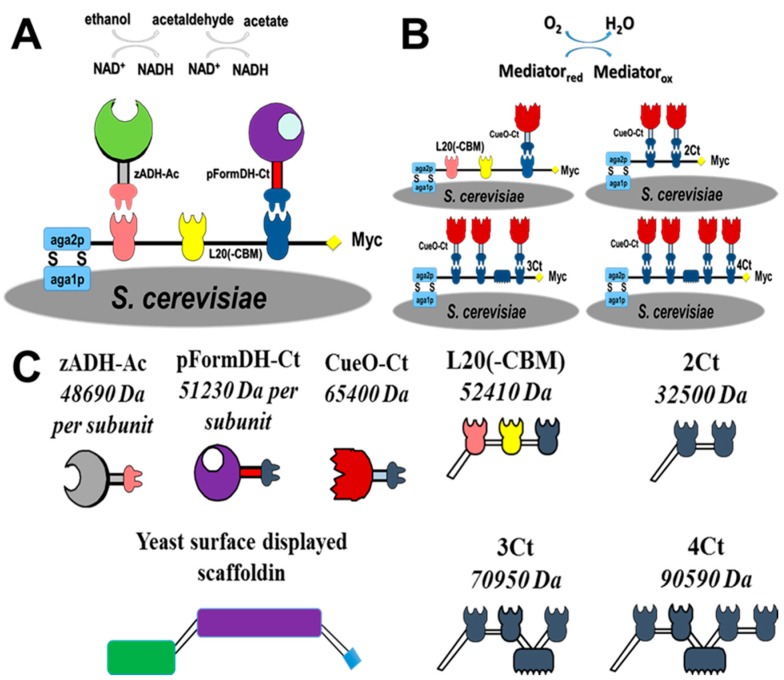
Schematic representation of (**A**) ethanol oxidation cascade displayed on the surface of *S. cerevisiae* using a chimeric scaffoldin, including dockerin-containing ADH and FormDH; (**B**) oxygen-reduction complexes displayed on *S. cerevisiae* using different scaffoldins, including one to four dockerin-containing copper oxidase copies; (**C**) all the enzymes and scaffoldins used in this study. Top row: dockerin-containing oxidoreductases. zADH-Ac: ADH from *Zymomonas mobilis* with an *Acetivibrio cellulolyticus* dockerin. pFormDH-Ct: *Pseudomonas putida* formaldehyde dehydrogenase with a *Clostridium thermocellum* dockerin. CueO-Ct: *E. coli* copper oxidase with a *C. thermocellum* dockerin. Bottom row from left to right: scaffoldins used in this study. 2Ct: di-valent scaffoldin comprising 2 *C. thermocellum* cohesins for CueO-Ct binding. 3Ct: tri-valent scaffoldin comprising 3 *C. thermocellum* cohesins for CueO-Ct binding. 4Ct: tetra-valent scaffoldin comprising 4 *C. thermocellum* cohesins for CueO-Ct binding. L20(-cellulose-binding module (CBM)): tri-valent scaffoldin, Scaf20L, comprising cohesins from *A. cellulolyticus*, *Bacteroides cellulosolvens*, and *C. thermocellum* but lacking the CBM for zADH-Ac and pFormDH-Ct; this construct was also used as a mono-valent CueO-Ct binding site. All the different scaffoldin constructs contain the Aga2p (depicted as a green rectangle) at the N-terminus and the c-myc (depicted as a blue square) at the C-terminus. A complete description of the system is given in the experimental section.

Depicted in Figure 1 is the electrosome that was designed for use both in an anode and a cathode compartment; in each compartment, the unique attributes of the cellulosome scaffoldin give a different advantage. In the anode (Figure 1A), the ethanol oxidation cascade consists of two enzymes, ADH and formaldehyde dehydrogenase (FormDH), both containing a different dockerin module of *Acetivibrio cellulolyticus* and of *Clostridium thermocellum*) *C. thermocellum*( (zADH-Ac and pFormDH-Ct), respectively, assembled on a ‘designer’-scaffoldin chimera displayed on the surface of *S. cerevisiae*. At the cathode (Figure 1B), copper oxidase (CueO) was selected for surface-display. CueO is a multi-copper oxidase enzyme expressed by *E. coli* that catalyzes the oxidation of Cu(I) ions coupled to oxygen reduction to water [37]. This enzyme is promiscuous and thus can oxidize different aromatic compounds, some of which can act as redox mediators in the cathode compartment of a biofuel cell. The dockerin module of *C. thermocellum* was fused to the enzyme (CueO-Ct), while, in parallel, we present the display of a mini-scaffoldin bearing one to four similar cohesins that can bind one to four copies of the dockerin-containing, oxygen-reducing enzyme CueO. The different constructs used for assembly are depicted in Figure 1C. We report the characterization of the dockerin-containing enzymes and their electrochemical activity using a diffusing redox mediator.

## 2. Results and Discussion

### 2.1. Choice of Enzymatic Cascade

The addition of a dockerin module to an enzyme may result in significant conformational changes as this module is relatively large (about 70 amino-acids long). Furthermore, when the native enzyme has a quaternary structure of several subunits, dockerin fusion may inhibit the formation of the quaternary structure and lead to a loss in activity. As a result, when selecting for enzymes, one should avoid choosing multimeric enzymes. In this work, due to the multimeric characteristics of most oxidoreductases, the enzymes were chosen after an analysis of their quaternary structure to prevent major loss in activity due to dockerin fusion. Hence, based on this criterion, pFormDH was selected to catalyze acetaldehyde oxidation in the cascade, even though it is more active towards formaldehyde. The ADH chosen for this study exhibits very high activity towards ethanol and negligible activity towards methanol. Hence, an ethanol oxidation cascade was chosen (Figure 1A).

### 2.2. Expression of Enzymes and Scaffoldin

All the scaffoldins and dockerin-containing enzymes are described schematically in Figure 1C. The YSD of the different scaffoldins was validated by flow cytometry using an anti c-myc antibody directed against a c-myc tag, which is part of the a-agglutinin display system (Supplementary Material Figure S1). In all of the experiments the YSD of the scaffoldins was compared to negative controls of non-modified yeast or yeasts known to display other enzymes on their surface (positive controls).

Colorimetric assays based on measuring the reduction of nicotine adenine di-nucleotide (NAD^+^) to NADH absorbance at 340 nm for zADH-Ac and pFormDH-Ct, were used to verify enzymatic activity of the dockerin-containing redox enzymes in bacterial lysates. Both enzymes have demonstrated activity compared to a negative control of native bacterial lysates. The activity was compared to the activity of a wild-type (WT), unmodified enzyme expressed in the same manner. A decrease in activity of approximately 33% and 75% was observed for zADH-Ac and pFormDH-Ct compared to their WT versions, respectively (Supplementary Material Figure S2A,B). This decrease in activity may have resulted from the addition of a relatively large dockerin domain to the enzyme, which may have reduced the expression levels and can affect affinity or substrate/co-factor accessibility to the enzyme’s active site. In addition, since the cascade is based on EtOH oxidation, the activity of the dockerin-containing pFormDH-Ct towards acetaldehyde was validated as well (Supplementary Material Figure S3). As both enzymes are multimers, the addition of the dockerin module may also interfere with inter-subunit binding. Although the observed loss in activity is quite significant, the dockerin-containing enzymes retain the ability to bind to scaffoldin proteins directly from bacterial lysates, avoiding a loss in protein yields, which are typical to complex purification processes. In addition, the total amount of active dockerin-containing enzyme is sufficient for binding to all available binding sites. The specificity of the enzymes towards their substrates was tested, and no activity was detected for zADH-Ac towards aldehydes or of pFormDH-Ct towards alcohols.

The activity of the dockerin-containing CueO-Ct was tested by measuring the change in absorbance at 430 nm following (orhto-phenilenediamine dihydrochloride) OPD oxidation (Supplementary Material Figure S4). The dockerin-containing CueO-Ct exhibited 10 times more activity in the lysate compared to a negative control consisting of native bacterial lysate. Native bacterial lysates are also expected to have OPD oxidation activity as a result of the presence of endogenous CueO, which is expressed when bacteria are grown in the presence of copper ions. WT CueO has demonstrated slightly higher activity compared to the dockerin-containing CueO. The smaller reduction in the enzyme activity of CueO compared to the other modified enzymes in this study, ca. only 20% loss of activity, is probably due to the structure of CueO; unlike the dehydrogenases, CueO is a monomer, which does not require an external co-factor, so the addition of the dockerin domain is less likely to interfere with its activity as long as the active site is accessible to the substrates. As with the dehydrogenases, with CueO as well, different expression levels may result from the dockerin addition.

**Figure 2:**
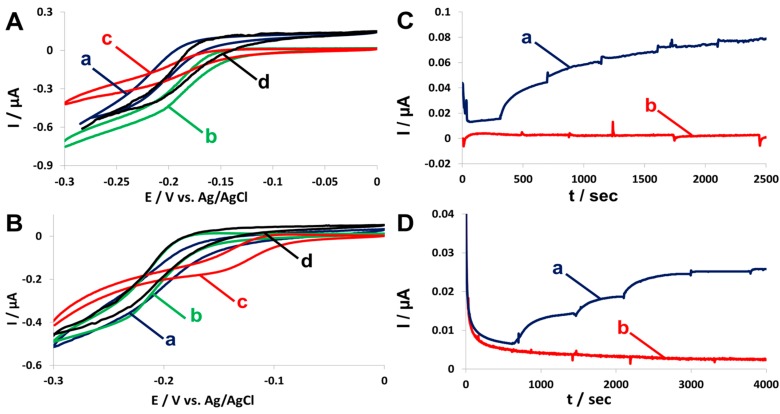
Electrochemical characterization of dockerin-containing enzymes. Cyclic voltammograms (CVs) of the electro-oxidation of (**A**) (a, dark blue) 5% *v*/*v* ethanol by a lysate of bacteria expressing zADH-Ac and (**B**) (a, dark blue) oxidation of 0.004% *v*/*v* formaldehyde by a lysate of bacteria expressing pFormDH-Ct ; (b, green) lysates of bacteria expressing the enzymes in the absence of the respective substrate; (c, red) lysates of native bacteria; (d, black) lysates of bacteria expressing the native enzymes, either zADH (**A**) or pFormDH (**B**), without a dockerin. Scan rate is 1 mV/s. Chronoamperometric measurements at 0.1 V vs. Ag/AgCl upon consecutive additions in each step of (**C**) 0.5% *v*/*v* EtOH or (**D**) 0.002% *v*/*v* formaldehyde to lysates of bacteria (a, dark blue) expressing a dockerin-containing zADH-Ac or pFormDH-Ct, respectively; (b, red) native bacterial lysates. Potential step: 0.1 V (vs. Ag/AgCl), Methylene blue 20 μM was used as a redox mediator and NAD^+^ 1.05 mM was used as a co-factor for both enzymes; reference electrode: Ag/AgCl.

Following the colorimetric enzymatic activity tests, enzymes in lysates were tested for their electrochemical and electrode communication abilities using a redox mediator by cyclic voltammetry and chronoamperometry. Demonstrating enzymes’ abilities to transfer electrons to and from electrodes is essential if these enzymes are to be used in a biofuel cell. Figure 2 shows an increase in the anodic current measured after the addition of bacterial lysate containing the dockerin-containing enzymes zADH-Ac (Figure 2A (a)) or pFormDH-Ct (Figure 2B (a)) in the presence of their respective substrates, ethanol or formaldehyde, respectively, and the cofactor NAD^+^, compared to the current measured in the presence of a native bacterial lysate (Figure 2A (b),B (b)). No catalytic current was observed in the absence of the respective enzymatic substrates (Figure 2A (c),B (c)). The lysates of bacteria over-expressing native enzymes were used as positive controls (Figure 2A (d),B (d)) and demonstrated electrocatalytic currents higher than those of the dockerin-containing enzymes which is in agreement with the results of the colorimetric assays. For both enzymes, methylene blue was used as a diffusive redox mediator. It can be observed that a relatively high reductive current is observed and is probably due to the NAD^+^ cofactor reduction. Although this does screen the effect of the bioelectrocatalytic oxidation of the analyte, the reduction of NAD^+^ could not be avoided and oxidation is still observed. In order to verify that bioelectrocatalysis indeed occurs, chronoamperometry measurements were conducted with the dockerin-containing zADH-Ac and pFormDH-Ct, compared to the negative control of native bacterial lysates. The incresae in the currents observed upon the addition of 0.5% *v*/*v* EtOH (Figure 2C (a)) or 0.002% *v*/*v* formaldehyde (Figure 2D (a)) to zADH-Ac and pFormDH-Ct-containing lysates, respectively, also demonstrates the electrochemical activity of these enzymes in comparison with the negative control lysates in which no increase in current was observed (Figure 2C (b),D (b)). The potential of the chronoamperometric measurements was chosen as it is more than 0.2 V higher than the onset bioelectrocatalytic oxidation wave potential (−0.1 V vs. Ag/AgCl); this way, the occurrence of oxidation in a daily changing living system such as the system described herein can be ensured.

**Figure 3:**
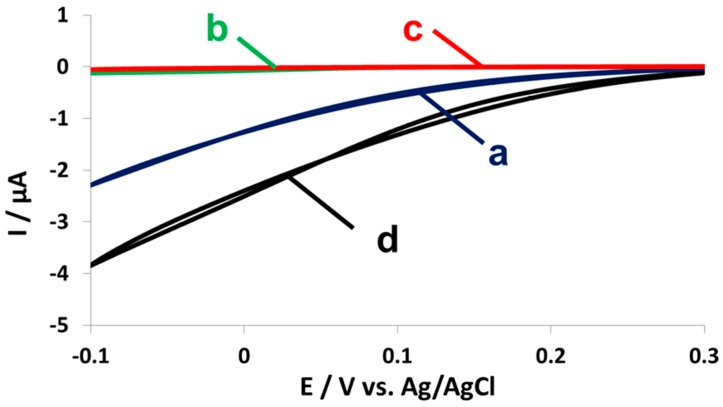
Electrochemical activity of the dockerin-containing CueO-Ct. CVs of the electroreduction of O_2_ in 0.1 M acetate by (a) lysates of bacteria expressing CueO-Ct; (b) no lysate; (c) lysates of native bacteria without enzyme over expression; (d) lysates of bacteria over-expressing CueO without a dockerin module. Cu^2+^ (10 μM) served as a redox mediator; reference electrode: Ag/AgCl; scan rate: 1 mV/s.

The electrochemical activity of the dockerin-containing CueO-Ct was validated using Cu(II) as the redox mediator in the presence of oxygen. Figure 3 shows the evolution of a cathodic current when a lysate of bacteria expressing CueO-Ct was added to the electrochemical cell (Figure 3 (a)) compared to the current measured with the addition of native bacterial lysate (Figure 3 (b)) and to the background current without any lysate (Figure 3 (c)). Due to the much higher cathodic current when electrocatalysis took place, it is hard to observe the reversible form of curve (Figure 3 (c)), but the typical reversible voltammograms of the mediator was observed with the middle of the wave at +0.05 V vs. Ag/AgCl. A WT enzyme was used as a positive control (Figure 3 (d)). The onset potential of the bio-electrocatalytic current is higher than that expected for mediated electron transfer but not as high as the potential expected from direct electron transfer of the enzyme (ca. 0.45 V vs. Ag/AgCl). This is probably due to the contribution of both processes to electron transfer, even though the enzyme has not been wired or physically attached to the surface of the electrode.

### 2.3. Enzyme Binding to Surface-Displayed Scaffoldins

**Figure 4:**
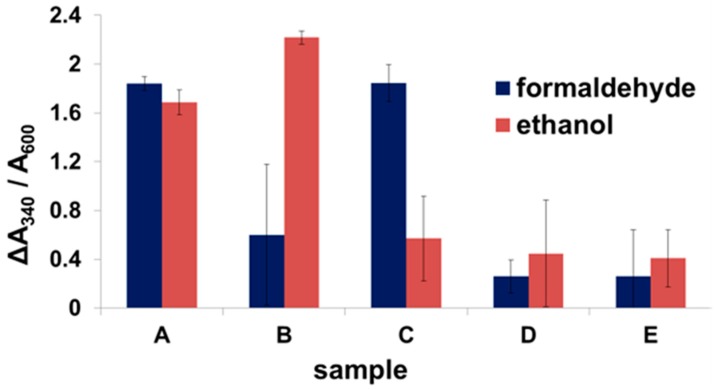
Binding of dockerin-containing enzymes to the scaffoldin-displayed yeast. Change in absorbance at 340 nm normalized to absorbance at 600 nm following the reduction of 2.1 mM NAD^+^ in the presence of either 1% *v*/*v* ethanol or 0.002% *v*/*v* formaldehyde in the presence of scaffoldin-displaying yeast incubated with (**A**) zADH-Ac and pFormDH-Ct in bacterial lysates; (**B**) zADH-Ac in bacterial lysate; (**C**) pFormDH-Ct in bacterial lysate; (**D**) native bacterial lysate; (**E**) native yeast cells incubated with both zADH-Ac and pFormDH-Ct in bacterial lysates. All assays were performed at 30 °C in Tris buffer 50 mM, pH 8.0.

To test the binding of the dockerin-containing enzymes to the scaffoldin displayed on yeast, colorimetric activity assays were performed using scaffoldin surface-displaying yeast cells incubated in bacterial lysates. Figure 4 shows the change in absorbance at 340 nm due to NAD^+^ reduction following the activity of each of the anodic enzymes with their respective substrates. Yeast suspended simultaneously in the lysates of bacteria expressing both enzymes displayed activity towards both substrates (Figure 4A). Yeast cells incubated with only zADH-Ac expressing lysates (Figure 4B) or pFormDH-Ct (Figure 4C) exhibited activity only towards their respective substrates. Scaffoldin-displaying yeast incubated with lysates of bacteria that did not express dockerin-containing enzymes did not show any activity (Figure 4D). In order to exclude any activity resulting from non-specifically bound enzymes, the yeast cells were washed after the binding assay, as described in the experimental section, and a control of native yeast cells, which were incubated with the dockerin-containing enzymes, was performed. No activity was measured for the control experiment (Figure 4E), which is a good indication that non-specific binding does not occur. Both control experiments indicate that the binding is specific and that activity does not stem from basal yeast activity. The activity of the two enzymes bound to yeast towards ethanol was not higher than that of zADH-Ac only, bound to yeast, probably due to the relatively low initial concentrations of acetaldehyde. As a result, NADH production was only due to zADH-Ac activity. As the pFormDH-Ct follows the same reaction, when acetaldehyde concentrations increase, more NADH accumulates; hence the increase in absorbance. These results suggest that, in fuel cells that operate for longer periods than the duration of the activity experiments, the effect of an enzymatic cascade will be observed to a higher extent. The change in absorbance in both assays was normalized to the absorbance at OD_600_ in order to exclude variation in yeast population density. The specificity of each enzyme in the cascade towards its substrate, observed in the assays performed using bacterial lysates, was maintained, even after enzyme binding to the scaffoldin. As the cohesin-dockerin interaction is species specific [38], each of the enzymes bound to its matching cohesin domain in the divalent chimeric scaffoldin. In the case of binding of zADH-Ac only, product inhibition may occur, but this inhibition may be avoided in the presence of the downstream enzyme. Yeast metabolism also contributes to further oxidation of acetaldehyde (EtOH oxidation product) but to a lesser extent than the yeast surface-displayed enzyme, pFormDH-Ct.

**Figure 5:**
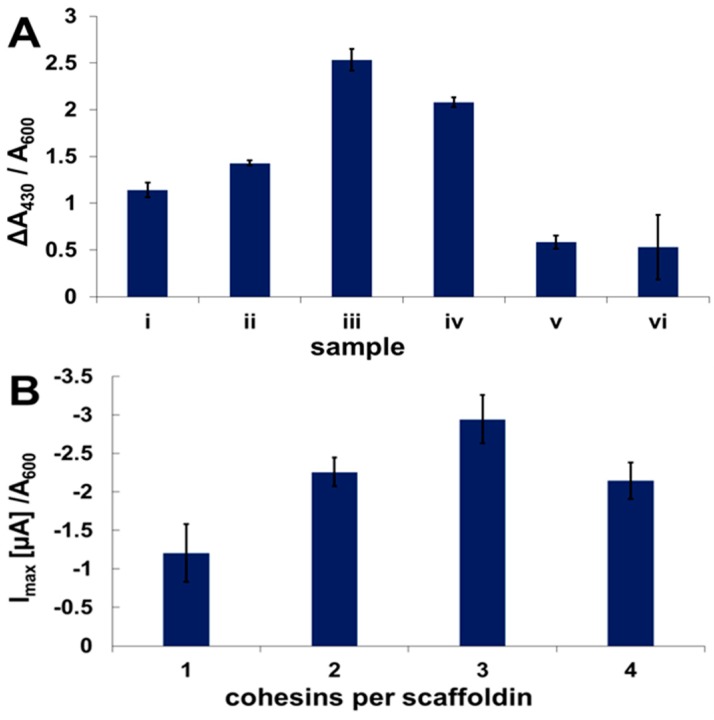
(**A**) Biochemical and (**B**) electrochemical activity of surface-displayed CueO-Ct normalized to OD_600_. (**A**) Change in absorbance at 430 nm normalized to 600 nm following the oxidation of OPD in the presence of yeast displaying a scaffoldin of (i) one; (ii) two; (iii) three; and (iv) four binding sites incubated with CueO-Ct expressing bacterial lysate; (v) WT yeast not displaying a scaffoldin incubated with CueO-Ct-expressing bacterial lysate; (vi) monovalent scaffoldin-displaying yeast incubated with a WT enzyme. (**B**) Maximum cathodic current achieved by CVs of yeast displaying scaffoldins of one to four binding sites incubated with CueO-Ct. The current is normalized to an absorbance at 600 nm. Cu^2+^ (10 μM) was used as a redox mediator; reference electrode: Ag/AgCl; scan rate: 1 mV/s.

For CueO-Ct, the change in absorbance at 430 nm due to OPD oxidation catalyzed by the enzyme was followed, and the dependence on the number of available binding sites was shown. Figure 5A shows the increase in absorbance upon binding of one to three enzyme copies to a monovalent, divalent, and trivalent mini-scaffoldin (Figure 5A (i–iii)). When a fourth binding site was added (tetravalent mini-scaffoldin), the activity decreased compared to the activity with three binding sites (Figure 5A (iv)). This suggests oxygen depletion due to the relatively high activity concentration on the yeast surface when binding sites for the enzyme are available. The specificity of binding was validated using different control samples of native yeast incubated with CueO-Ct (Figure 5A (v)) or of scaffoldin-displaying yeast incubated with a WT enzyme that cannot bind yeast (Figure 5A (vi)), which demonstrates that there is no activity that stems from non-specifically bound enzymes. In both control experiments, the activity was significantly lower, reflecting only background yeast activity.

Electrochemical activity was measured for the same samples. Figure 5B shows an increase in the cathodic peak current when mini-scaffoldins with one to four binding sites were used, after suspending yeast in CueO-Ct-containing lysate. The currents were normalized to the OD_600_ in order to neutralize the increase in absorbance stemming from cell growth and cell division from the number of active enzyme copy numbers. From Figure 5B, it can be observed that, when using between one and three binding sites, the current increases as expected by ca. 1 μA with each binding site added. However, as is evident from the colorimetric assay, upon binding of four enzymes there is a decrease in current, showing a current that is similar to that observed for two binding sites. Again, we attribute the reduction in currents with a fourth binding site to oxygen depletion at the yeast surface.

### 2.4. Fuel Cell Assembly and Characterization

**Figure 6:**
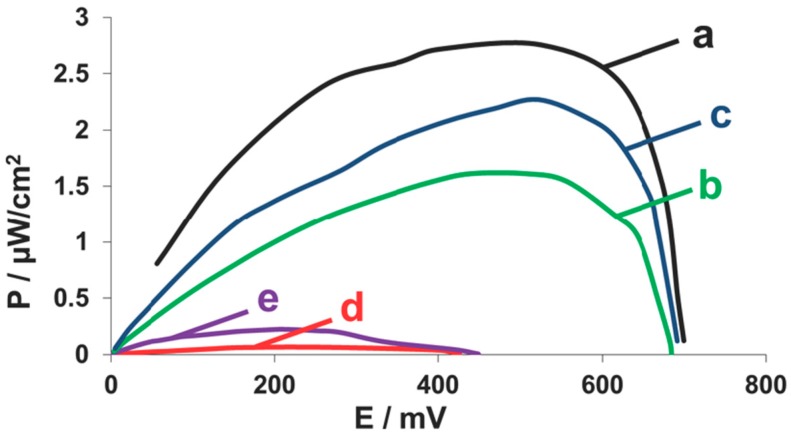
Performance of yeast surface-displayed enzyme cascade. Power output curves of anodes of scaffoldin-displaying yeast that were incubated with (a) a mixture of zADH-Ac- and pFormDH-Ct-expressing bacterial lysates; (b) zADH-Ac-expressing bacterial lysate; (c) pFormDH-Ct-expressing bacterial lysate; (d) native bacterial lysate; (e) native yeast incubated with both zADH-Ac- and pFormDH-Ct-expressing bacteria lysates. EtOH 2% *v*/*v* as fuel, NAD^+^ 1.05 mM and 1 mM MB have been used as the enzyme cofactor and redox mediator, respectively.

Following the demonstration of electrochemical activity, fuel cells were assembled to demonstrate the activity of the enzymes bound to scaffoldins displayed on the surface of yeast. Figure 6 shows the polarization and power output curves of the cells using ethanol as fuel when using yeast displaying a cascade of two enzymes (Figure 6 (a)) compared to cells of just zADH-Ac (Figure 6 (b)) or pFormDH-Ct (Figure 6 (c)). As expected, the maximum power output of 2.7 ± 0.1 μW·cm^−2^ was measured with a cascade-displaying yeast. The higher power output of the yeast displaying only the second pFormDH-Ct, 2.2 ± 0.3 μW·cm^−2^, compared to those expressing only the first zADH-Ac enzyme, 1.6 ± 0.2 μW·cm^−2^, can be explained by the expression of the native yeast ADH, which catalyzes the first reaction in the cascade, which is then followed by the reaction catalyzed by the scaffoldin-bound pFormDH-Ct. When only the zADH-Ac is bound, this enzyme contributes to the cell performance, but the following reaction does not occur. The control samples of yeast displaying a scaffoldin without bound enzymes (Figure 6 (d)) or native yeast cells incubated with dockerin-containing enzymes (Figure 6 (e)) did not demonstrate any electrochemical activity. In all cases, the observed performance was lower than that observed for the cascade. In addition, the background current without any addition of yeast was very low. The power output was also plotted versus the current density, showing better performance of the cascade (Supplementary Material, Figure S5). Comparing to the previously presented hybrid cells of one enzyme [5,13], an improvement in power output is observed. A control cell without yeast was used to test the background currents and has shown very low currents (not shown), lower than observed in Figure 6 (d,e), depicting the negative control samples.

**Figure 7:**
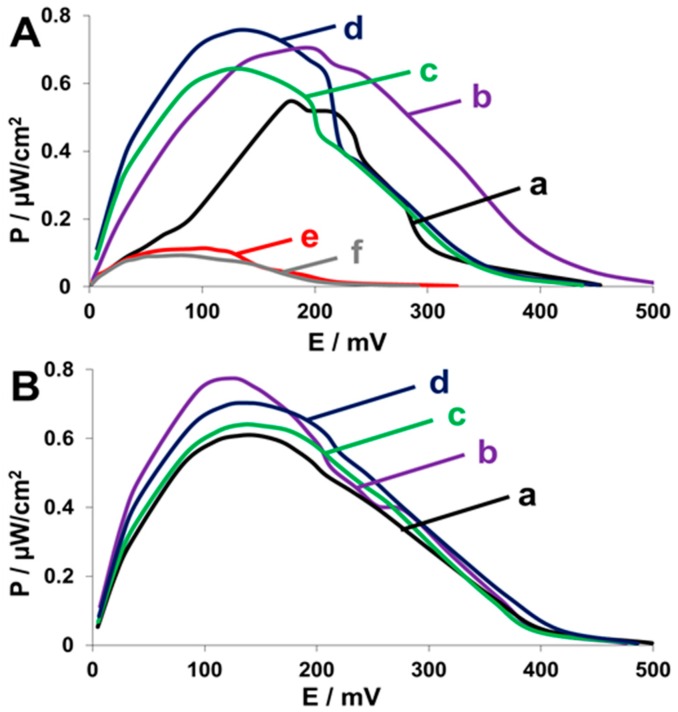
Performance of biocathodes comprised of yeast containing increasing copy number of surface-displayed CueO. Power output curves for (**A**) different scaffoldin-displaying yeast with (a) monovalent; (b) bivalent; (c) trivalent; and (d) tetravalent scaffoldins with CueO-Ct; (e) native yeast with CueO-Ct; (f) monovalent scaffoldin-bearing yeast incubated with native CueO; (**B**) Power output curves of cells with antimycin A and with (a) monovalent; (b) divalent; (c) trivalent; (d) tetravalent mini-scaffoldins. Air was purged to the cells, Cu^2+^ 25 μM as redox mediator and enzyme cofactor.

Hybrid biocathodes, containing yeast displaying an increasing number of CueO copies per surface-displayed scaffoldin, were assembled. These modified scaffoldin-bearing yeast were used as catalysts in a hybrid cathode to which air was purged in order to increase oxygen levels. Figure 7A shows the power output curves of the different cells. Unlike the trend observed in the biochemical and electrochemical assays, the highest power output of ca. 0.76 ± 0.08 µW·cm^−2^ was observed with yeast displaying four binding sites for tetra-valent mini-scaffoldin (Figure 7A (d)). Yeast displaying two binding sites (di-valent mini-scaffoldin) exhibited the second highest power output of 0.70 ± 0.05 µW·cm^−2^, but that was observed at higher voltage, indicating a higher internal resistance compared to that of all the other cells (Figure 7A (b)). The lowest power output for scaffoldin-displaying yeast was achieved with the yeast displaying a scaffoldin with only a single binding site (mono-valent mini-scaffoldin), 0.054 ± 0.06 µW·cm^−2^ (Figure 7A (a)). The difference between the fuel cells and the different assays presented herein can be explained by the fact that the yeast cells are exposed to different conditions in the fuel cells, relative to those in the electrochemical cell or the biochemical activity tests. The biochemical activity assay was conducted shortly upon binding between enzymes and the scaffoldin in a fresh buffer, while the fuel cells are more complex living systems that are allowed to stabilize after assembly and before characterization. After an equilibration period, the oxygen concentrations in the medium may be lower than their initial concentrations. However, diffusion limitation is lower as well since there is no immediate demand for high oxygen concentration as in the CV measurements. Thus, the expected increase in power output, stemming from a high copy number of displayed enzymes, is observed. The purging of air to the assembled fuel cells, which was not performed in the activity assays, further improves mass transfer and oxygen availability to the enzymes. As expected, control populations of yeast that cannot bind CueO-Ct (Figure 7A (e)) or scaffoldin-displaying yeast that have been incubated with the native enzyme (Figure 7A (f)), which cannot bind, exhibited significantly lower power outputs of 0.11 ± 0.06 µW·cm^−2^ and 0.09 ± 0.06 µW·cm^−2^, respectively, representing yeast basal activity, which is close to the error of the measurements. A control biofuel cell without yeast was used to test the background currents and has shown very low currents (not shown), lower than those observed in Figure 7A (e,f) negative control samples.

The hybrid biocathodes performed well without the need to inhibit yeast aerobic respiration, thus indicating that the competitive inhibition effect resulting from oxygen consumption by yeast was overcome by the high density of oxygen-reducing enzymes on the yeast surface compared to previously reported biocathodes, in which the effect resulted in a significant loss in power output [8]. In order to validate this finding, antimycin A, which is an inhibitor of yeast aerobic respiration [36], was added to the different cathodes, and the cathodes’ performance was compared to that of cathodes in the absence of antimycin A. We expected that as copy number per cell increases, the competitive inhibition effect will decrease. Thus, the effect of antimycin A addition on performance should be the largest for monovalent scaffoldins. Figure 7B shows that while for one and two copies of CueO-Ct bound to each scaffoldin (Figure 7B (a,b)), the performance of the cells improved and higher maximum power outputs of 0.61 ± 0.05 µW·cm^−2^ and 0.78 ± 0.02 µW·cm^−2^ (for mono-valent and di-valent scaffoldin, respectively) were measured, in comparison with 0.54 ± 0.03 µW·cm^−2^ and 0.70 ± 0.03 µW·cm^−2^, respectively, without antimycin A. For the higher copy numbers such as tri-valent and tetra-valent scaffoldins, the power output was similar to that achieved without antimycin A, demonstrating the same results within the similar measured error (Figure 7B (c,d)). These results suggest that competitive inhibition can be overcome without the addition of antimycin A, which, for long-term usage, is not a sustainable approach in MFCs. In order to fully overcome oxygen competition, more complexed scaffoldin proteins, which consist of more cohesin modules, should be surface-displayed. The use of higher redox potential laccases and improved electron transfer or wiring will further improve fuel cell performance. In addition, CueO may be engineered for improved performance in biofuel cell devices. The power outputs were also plotted against the current density (Supplementary Material Figure S6) and further demonstrated the improvement in cell performance upon the addition of CueO copies per yeast cell and that, in the higher copy number, the antimycin A is not contributing to the cell performance, unlike in the low copy number.

The characterization of each compartment (anode and cathode) presented herein was performed separately in order to avoid the influence of the other compartment on characterization. Furthermore, the assembly of a two-compartment hybrid cell requires also the presence of a proton-exchange membrane, which affects cell performance due to the introduction of higher resistances. Power output losses that might occur as a result of cell architecture, which is not relevant to the systems presented in this study, are also excluded in a semi-biofuel cell configuration. The use of a potentiostatically controlled electrodes in a membraneless assembly may affect the system. Therefore the counter electrode in the three-electrode system used herein had a higher surface area, as described in the experimental section. In the case of the hybrid anode, setting the potential of the cathode to a higher potential may result in the oxidation of the redox mediator. However, the usage of a membraneless assembly has given us an advantage in terms of internal resistance, which is crucial in the simple assembly used for the proof of concept. Different control cells, including a cell of background current, were used to demonstrate that the differences in performance are a result of the different yeast populations that were used in this study.

## 3. Conclusions

We have designed a novel yeast surface-display system displaying different scaffoldin proteins in order to improve the performance of hybrid biofuel cells. This approach enabled us to display a cascade of ethanol oxidation enzymes bound to a chimeric scaffoldin in the anode, which demonstrated higher performance than a cell displaying only a single enzyme. The system has demonstrated comparable performance to that of our yeast surface-displayed cellobiose dehydrogenase (CDH) system [13]. Taking into consideration that, in the CDH system, four electrons oxidation occurs, which is similar to the number of electrons resulting from the ethanol oxidation cascade presented herein. This performance is significantly higher than our first example of a hybrid biofuel cell with a yeast surface-display of glucose oxidase [5]. The main advantage of the cascade assembly is that it is not limited to one enzyme reaction, meaning that, by further addition of enzymes, wiring and surface coverage improvement can be achieved in follow-up studies.

In addition, this approach allowed us to use crude lysates. Thus, we were able to avoid the purification of two different enzymes. In the cathode, we displayed the oxygen-reducing enzyme CueO in a sequential addition of copy-numbers; from one enzyme bound to a mono-valent scaffoldin to up to four enzymes bound to a tetra-valent mini-scaffoldin in a single cell. This approach resulted in reduced competitive inhibition effects resulting from oxygen consumption by yeast, thereby avoiding the need for the inhibition of yeast aerobic respiration using antibiotics or other inhibitors. It should be noted that the power output demonstrated in the fuel cells presented, is low compared to previously reported enzymatic biofuel cells systems. However, the system presented herein is a proof of concept, demonstrating that, with further engineering of the enzymatic cascades and scaffoldin, as well as biofuel cell’s architecture and engineering, much higher power outputs may be achieved while exploiting the advantages of the hybrid fuel cell, which is a living system that does not require enzyme purification and is more suitable for long-term operation.

This approach paves the way for the assembly of more intricate cascades using different enzymes and larger scaffoldins such as the recently described adaptor scaffoldins [28], which will further improve hybrid cell performance. Considering that mediated electron transfer is less suitable for the long-term operation of the system, further studies require the engineering of systems that are able to perform direct electron transfer. The demonstration of Amir et al. [9] of the site-specific wiring of an enzyme can be used. In addition, the ability of CueO to perform through direct electron transfer can further improve fuel cell performance. As the structure of the enzyme influences its activity when fused to a dockerin module, this should be considered when choosing enzymes for such an application. The improvement of an enzymatic cascade and substrate channeling strategies should be performed hand in hand with improvements in electrode material as well as enzyme/microorganism-immobilization approaches.

## 4. Materials and Methods

### 4.1. Strains and Constructs

The genes encoding dockerins of *Acetivibrio cellulolyticus* (dockerin from ScaB scaffoldin strain ATCC 33288) and *Clostridium thermocellum* (dockerin from Cel48S strain ATCC 27405) were cloned and ligated to the C-terminus of *Zymomonas mobilis* alcohol dehydrogenase and to *Pseudomonas putida* formaldehyde dehydrogenase (zADH-Ac and pFormDH-Ct, respectively) by standard methods [39]. The dockerin module of *C. thermocellum* was also ligated to the C-terminus of CueO (CueO-Ct) of *E. coli* (all the sequences are listed in the supplementary material (SI) section). All the dockerin-containing enzymes encoding genes have been cloned into the pET15b vector for expression in *E. coli*, yielding the pET15b-zADH-Ac, pET15b-pFormDH-Ct, and pET15b-CueO-Ct vectors. For controls, the genes encoding the native enzymes without an appended dockerin module were also cloned in the same vector, yielding plasmids pET15b-zADH, pET15b-pFormDH, and pET15b-CueO. All the chemicals used in this study are detailed in the SI section.

### 4.2. Protein Expression

For protein expression, a 10-mL culture of *E. coli* bacteria was grown overnight at 37 °C in standard Luria-Bertani broth with carbenicillin (Chem-Impex International, Wood Dale, IL, USA) at a concentration of 100 μg/mL. A volume of 1.0 mL of the culture was used to inoculate a 100-mL culture in the same medium containing carbenicillin at the same concentration. The culture was incubated at 37 °C until it reached an optical density (O.D.) of 0.5 at 600 nm. Then 1 mM Isopropyl β-d-1-thiogalactopyranoside (IPTG, Inalco, San Luis Obispo, CA, USA) was added to induce protein expression, followed by the overnight incubation of the cultures at 20 °C. The bacteria cultures were lysed by sonication, and the lysates containing the proteins were separated by precipitation. The cells were lysed in 50 mM Tris buffer, pH 8.0, containing 1 mM CaCl_2_. For cells expressing CueO, lysis was performed using 0.1 M acetate buffer, pH 5.0, containing 1 mM CaCl_2_ (for proper dockerin folding) and 800 μM CuSO_4_. For all the lysates preparations, the bacterial cultures were diluted to the same optical density of 1.5 in 1:20 dilution in the binding buffer in order to ensure that the same number of bacteria was lysed from each culture and to measure the differences resulting from enzyme expression and activity levels.

### 4.3. Enzyme Activity Assays

The activity of the enzymes was tested in their respective lysates. The activity of zADH and pFormDH was tested by adding the bacterial lysates and following the change in absorbance at 340 nm due to the reduction of β-Nicotinamide adenine dinucleotide (NAD^+^, Sigma-Aldrich, Rehovot, Israel), which is a co-factor of both enzymes. For the zADH assay, 1–5% *v*/*v* EtOH was used as a substrate, and, for the pFormDH assay, 0.002% *v*/*v* formaldehyde or 50 mM acetaldehyde was used. A concentration of 1.05 mM of NAD^+^ was added to both enzymatic assays. All assays were performed in 50 mM Tris buffer at pH 8.0 containing 1 mM CaCl_2_ (for proper cohesin-dockerin interaction).

The activity of CueO was tested by following the change in absorbance at 430 nm due to the oxidation of 3.7 mM *o*-phenylenediamine (OPD), catalyzed by CueO. A SigmaFast OPD kit (Sigma-Aldrich, Rehovot, Israel) was used while bacterial lysates expressing CueO were added to the kit reaction mixture. The assay was performed at 0.1 M acetate buffer pH 5.0, containing 1 mM CaCl_2_ and 800 μM CuSO_4_.

### 4.4. Construction of YSD of Chimeric Scaffoldins

Genes encoding monovalent scaffoldins (one cohesin module) from *A. cellulolyticus* (cohesin 3 from the ScaC scaffoldin), *Bacteroides cellulosolvens* (cohesin 3 from ScaB), and *C. thermocellum* (cohesin 3 from the CipA scaffoldin) were cloned into the pCTCON a-agglutinin YSD vector. In the process, the cellulose binding module (CBM) of the scaffoldin was removed, yielding vector pCTL20 (-CBM). The *B. cellulosolvens* cohesin was inserted to enable the binding of a third enzyme in future studies. Scaffoldins with two, three, and four cohesins of *C. thermocellum* (cohesins 8 and 9 from CipA or cohesins 1, 2 and 3 or cohesins 2, 3, 4 and 5, respectively) were cloned into the same vector, yielding vectors pCT2Ct, pCT3Ct, and pCT4Ct, respectively. The cloning was performed between the Aga2p, which enables the binding of the YSD system to the yeast cell, and the c-myc tag, which can be used for YSD validation [7]. The genes were transformed to the EBY100 *S. cerevisiae* yeast strain. The expression of the YSD system was performed as described earlier [5,12]. YSD was validated by flow cytometry using the c-myc tag of the YSD system, as described and shown in detail in the SI section.

### 4.5. Enzyme Binding to Scaffoldin

For enzyme binding, 2.0 mL of yeast cells displaying scaffoldin, for which absorbance at a wavelength of 600 nm was 1.0 (full description of the surface-display process can be found in the SI section), were incubated with bacterial lysates containing the expressed enzymes at room temperature for 1 h. 1.0 mL of the bacterial lysates were used for the binding, which was performed in a final volume of 15 mL. As a binding buffer, 50 mM Tris buffer at pH 8.0 with 1 mM CaCl_2_ was used. Upon binding, the yeast cells were precipitated, and binding was repeated using fresh lysate. After the second binding cycle, the yeast cells were washed four times in the buffer to remove non-specifically bound enzymes. For the CueO-Ct binding, the yeast cells were suspended in 0.1 M acetate buffer pH 5.0 containing 1 mM CaCl_2_ after the last wash. Following binding, the yeast calls were resuspended in 2.0 mL of buffer.

### 4.6. Cyclic Voltammetry (CV) and Chronoamperometry (CA)

A standard three electrode electrochemical cell was used. 0.9 mm diameter graphite rods (Pilot, Tokyo, Japan) served as both working and counter electrodes, and Ag/AgCl was used as the reference electrode (ALS, Tokyo, Japan). The assembly was designed so the counter electrode would have a surface area 10 times greater than that of the working electrode. All measurements were conducted using a PalmSens potentiostat (PalmSense BV, Houten, The Netherlands). For CV measurements, 100 μL of bacterial lysates prepared as described above were added to the electrochemical cell containing the enzyme’s substrate and cofactors. For zADH and pFormDH, 20 μM methylene blue (MB), as a redox mediator, and 1.05 mM NAD^+^ were added. For the CueO, 20 μM CuSO_4_ was added as the redox mediator, which also functions as the enzyme cofactor. The scan rate was 1 mV/s in all the measurements in a potential range of −0.3–0 V versus Ag/AgCl for the zADH and pFormDH or −0.1–+0.3 V versus Ag/AgCl for CueO. The final reaction volume was 2.0 mL. The CV measurements with yeast were performed under the same conditions, substituting the bacteria lysate with 200 μL of enzyme-bound yeast suspension. CA was performed to demonstrate the electrochemical activity of the anodic cascade dockerin-containing enzymes at a potential of +0.1 V vs. Ag/AgCl. The same volume of bacterial lysates as in the CV, 100 μL, was added to a final volume of 2.0 mL. 20 μM MB served as a redox mediator and 1.05 mM NAD^+^ as the cofactor of both enzymes. 0.5% *v*/*v* of EtOH or 0.002% *v*/*v* of formaldehyde were added. The bacterial lysates of native bacteria were used as a negative control. All electrochemical measurements have been performed in triplicates and have given stable and similar curves.

### 4.7. Biofuel-Cell Assembly and Characterization

The fuel cell assembly was performed in 50 mL glass vials. For the cascade assay, 5.0 mL of bound yeast was precipitated and added to the cell containing 1.05 mM NAD^+^ and 1 mM MB. Prior to the addition of yeast, Argon was purged to the medium for 1 h. 2% *v*/*v* EtOH was added following the addition of yeast, and the cells were sealed using Parafilm (Bemis, Oshkosh, WI, USA). The total volume of the cells was 10 mL. A potentiostatically controlled cathode was set to +0.5 V vs. Ag/AgCl using a PalmSens MultiEmStat potentiostat (PalmSens BV, Houten, The Netherlands), as performed by Xia et al. [11]. For the CueO-based fuel cells, the CueO-Ct bound yeast were added to a cell containing 25 μM CuSO_4_ and the antibiotics antimycin A (10 μM). Air was continuously purged to the fuel-cells. A potentiostatically controlled anode set to −0.2 V versus Ag/AgCl was used. In all experiments, the cells were left to stabilize overnight, following fuel cell assembly, before characterization was performed. The characterization of fuel cell performance was done by measuring the voltage of the cells under variable external loads. A background current cell was used as a negative control for all fuel cell experiments and did not contain any yeast. Graphite rods of 5 mm diameter served as both anodes and cathodes. The counter electrode that served for the potentiostatically controlled electrode was of a larger surface area, as described for the CV and CA measurements.

## Supplementary Materials

The following are available online at http://www.mdpi.com/2079-4991/7/7/153/s1, Table S1: Strains and plasmids used in this study, Table S2: scaffoldin gene sequences, Table S3: dockerin-containing genes sequences, Figure S1: FACS analysis, Figures S2–S4: enzymatic activity assays in lysate, Figure S5: power output vs. current density curves for the anodic cascade biofuel cells, Figure S6: power output vs. current density curves for the biocathodes biofuel cells.

## Abbreviations

The following abbreviations are used in the text:

MFC	microbial fuel cell
YSD	yeast surface-display
ADH	alcohol dehydrogenase
AldDH	aldehyde dehydrogenase
FormDH	formaldehyde dehydrogenase
GOx	glucose oxidase
zADH	*Zymomonas mobilis* alcohol dehydrogenase
pFormDH	*Pseudomonas putida* formaldehyde dehydrogenase
CueO	*Escherichia coli* copper oxidase
BOD	bilirubin oxidase
Ac	*Acetivibrio cellulolyticus*
Ct	*Clostridium thermocellum*
CBM	Cellulose-binding module
CV	cyclic voltammetry
CA	chronoamperometry
MB	methylene blue
NAD^+^	β-Nicotinamide adenine dinucleotide
OPD	*o*-phenylenediamine dihydrochloride
DMSO	dimethyl sulfoxide
IPTG	Isopropyl β-d-1-thiogalactopyranoside
PEG	polyethylene glycol
WT	wild type
EtOH	ethanol
MeOH	methanol
FACS	Fluorescence Activated Cell Sorting
