# Supplementary material for: The Electrosome: A Surface-Displayed Enzymatic Cascade in a Biofuel Cell’s Anode and a High-Density Surface-Displayed Biocathodic Enzyme
Source: Nanomaterials (Basel). 2017 Jun 23;7(7):153. doi: 10.3390/nano7070153 (PMC5535219; doi:10.3390/nano7070153)
Supplement: Supplementary file 1 [file nanomaterials-07-00153-s001.pdf]

Supporting Information

The Electrosome: A Surface Displayed Enzymatic Cascade in a Biofuel Cell's anode and a High Density Surface Displayed Biocathodic Enzyme

Alon Szczupak[a], Dror Aizik[a], Sarah Moraïs[b], Yael Vazana[b], Yoav Barak[c], Edward A. Bayer[b] and Lital Alfonta[a*]

[a]Department of Life Sciences and the Ilse Katz Institute for Nanoscale Science and Technology, P.O.Box 653, Beer-Sheva,8410501 Israel [b]Department of Biomolecular Sciences, Weizmann Institute of Science, 234 Herzl St. P.O.Box 26, Rehovot 7610001, Israel. [c]Department of Chemical Research Support, Weizmann Institute of Science, 234 Herzl St. P.O.Box 26, Rehovot 7610001, Israel.

Materials and methods

Materials. o-phenylenediamine dihydrochloride (SIGMA *FAS*T^{TM} OPD), $CaCl_2$, acetaldehyde, sodium acetate, ferric citrate, polyethylene glycol (PEG), lithium acetate, $CuSO_4$, albumin from egg yolk, Tris-Base, antimycin A, δ-aminolevulinic acid, dimethyl sulfoxide (DMSO), methylene blue (MB), D-galactose, D-glucose (All from Sigma-Aldrich, Rehovot, Israel); Isopropyl β-D-1-thiogalactopyranoside (IPTG, Inalco, San Luis Obispo, CA, USA); formaldehyde, $NaH_2PO_4 \cdot H_2O$ (Thermo Fisher Scientific, Waltham, MA, USA); ethanol (Carlo Ebra, Val de Reuil, France); herring sperm DNA (Promega, Madison, WI, USA); Na_2HPO_4 (Alfa Aeser, Ward Hill, MA, USA); carbenicillin (Chem-Impex International, Wood Dale, IL, USA); NaCl (Daejung, Gyeonggi-do, Korea); bacto peptone, bacto casamino acids, difco yeast nitrogen base without amino acids Tryptone, yeast extract (BD, Franklin Lakes, NJ, USA); mouse anti-c-Myc IgG1, goat anti mouse PE conjugate (Abcam, Cambridge, UK), 0.9mm graphite rods (Pilot, Tokyo, Japan); Kapa HiFi PCR Kit (Kapa Biosystems, Woburn, MA, USA); RE-1B Ag/AgCl reference electrode (ALS, Tokyo, Japan).

Plasmids. Plasmids that were used or constructed in this study are listed in the following table S1.

Plasmid	Characteristics	Source or reference
**Plasmids**		
pCTCON	YSD vector	[1]
PCGA	YSD vector expressing glucoamilase	[2]

pCTL20(-CBM)	YSD vector expressing scaffoldin chimera	This research
pCT2Ct	YSD vector expressing 2 cohesins scaffoldin	This research
pCT3Ct	YSD vector expressing 3 cohesins scaffoldin	This research
pCT4Ct	YSD vector expressing 4 cohesins scaffoldin	This research
pET15b	*E. coli* expression vector	commercial
pET15b-zADH-Ac	Dockerin-containing zADH expression vector	This research
pET15b-pFormDH-Ct	Dockerin-containing pFormDH expression vector	This research
pET15b-CueO-Ct	Dockerin-containing CueO expression vector	This research
pET15b-zADH	Wild-type zADH expression vector	[3]
pET15b-pFormDH	Wild-type pFormDH expression vector	This research
pET15b-CueO-Ct	Wild-type CueO expression vector	This research

Table S1. Strains and plasmids used

Strains and media. *Escherichia coli* DH5α and BL21 strains were used as host strains for recombinant DNA amplification and CueO expression. *Saccharomyces cerevisiae* EBY100[1] was used for cell-surface expression.

E. coli were grown in standard Luria-Bertani broth (LB) medium containing 100 mg/L carbenicillin when necessary, at 37°C with continuous shaking at 250 rpm. EBY100 yeast were grown at 30°C either in YPD media (20g/l glucose, 20g/L peptone, 20g/L yeast extract), or on SC ura$^-$ plates[4]. Transformed yeast were grown at 30°C either media composed of 20g/L glucose, 6.7g/L yeast nitrogen base without amino acids, 5g/L Bacto casamino acids, 6.8g/L $Na_2HPO_4{\cdot}12H_2O$, 9.6g/L $NaH_2PO_4{\cdot}H_2O$ (SDCAA) or on SC ura$^-$ trp$^-$ plates. Induction media for transformed yeast comprised 90% SGCAA media which is similar to SDCAA, but galactose was used instead of glucose, 10% SDCAA media, 3.6mM δ-aminolevulinic acid and 0.2mM ferric citrate. Induction conditions were incubation for at least 18hr at 20°C, 30°C, or 37°C with continuous shaking at 250rpm.

Construction of plasmids for cell surface display of scaffoldin proteins. For the 2 to 4 cohesins scaffoldin, a gene encoding for the CipA scaffoldin protein of *Clostridium thermocellum* ATTC 27405 has been used. The genes encoding the cohesins 8 and 9 from CipA, or cohesins 1, 2 and 3 or cohesins or cohesins 2, 3, 4 and 5 have been amplified and extracted

by standard PCR. A *Nhe*I restriction site was added to the 5' end of the gene and a *Bam*HI site to the 3' end and the genes have been cloned into the *Nhe*I-*Bam*HI sites in the pCTCON vector. The procedure has yielded the pCT2Ct, pCT3Ct and pCT4Ct vectors. For the one binding site scaffoldin a gene encoding a scaffoldin chimera containing one cohesin module from *C. thermocellum* (cohesin 3 from CipA scaffoldin), *Acetovibrio cellulolyticus* (cohesin 3 from ScaC scaffoldin) and *Bacteroides cellulosolvens*, and a cellulose binding module (CBM)[5] have been extracted and cloned to the pCTCON vector in the same manner. The CBM has been removed in the process yielding the pCTL20(-CBM) vector. All genes have been donated by Prof. Edward A. Bayer. Gene sequences are listed in the following table S2.

Gene	Sequence
L20(-CBM)	ggatccgatttacaggttgacattggaagtactagtggaaaagcaggtagtgttgttagtgtacctataac<br>atttactaatgtacctaaatcaggtatctatgctctaagttttagaacaaatttcgacccacaaaaggtaact<br>gtagcaagtatagatgctggctcactgattgaaaatgcttctgattttactacttattataataatgaaaatgg<br>ttttgcatcaatgacgtttgaagccccagttgatagagctagaatcatagatagtgatggtgtatttgcaac<br>cattaactttaaagttagtgatagtgccaaagtaggtgaactttacaatattactactaatagtgcatatactt<br>cattctattattctggaactgatgaaatcaaaaatgttgtttacaatgatggaaaaattgaggtaattgcaagt<br>cctaccccgacgcaatcagccactccaacggtaactccttcagccaccgcgacgcctacccagagtgc<br>tacgccgactgtaacgccaagttcaccaggaaataaaatgaaaattcaaattggtgatgtaaaagctaat<br>cagggagatacagttatagtacctataactttcaatgaagttcctgtaatgggtgttaataactgtaatttcac<br>tttagcttatgacaaaaatattatggaatttatctctgctgatgcaggtgatattgtaacattgccaatggcta<br>actatagctacaatatgccatctgatgggctagtaaaatttttatataatgatcaagctcaaggtgcaatgtc<br>aataaaagaagatggtacttttgctaatgttaaatttaaaattaagcagagtgccgcatttgggaaatattca<br>gtaggcatcaaagcaattggttcaatttccgctttaagcaatagtaagttaatacctattgaatcaatatttaa<br>agatggaagcattactgtaactaatacgccgaccaatactatcagtgttactccgacaaacaattcgactc<br>ctacgaataacagtacgccaaagccaaacccgttatccgacggtgtggtagtagaaattggcaaagtta<br>cgggatctgttggaactacagttgaaatacctgtatatttcagaggagttccatccaaaggaatagcaaac<br>tgcgactttgtgttcagatatgatccgaatgtattggaaattatagggatagatcccggagacataatagtt<br>gacccgaatcctaccaagagctttgatactgcaatatatcctgacagaaagataatagtattcctgtttgcg<br>gaagacagcggaacaggagcgtatgcaataactaaagacggagtatttgcaaaaataagagcaactgt<br>aaaatcaagtgctccgggctatattactttcgacgaagtaggtggatttgcagataatgacctggtagaac<br>agaaggtatcatttatagacggtggtgttaacgttggcaatgcaaca
2Ct	gtaaggattaaagtggacacagtaaatgcaaaaccgggagacacagtaagaatacctgtaagattcag<br>cggtataccatccaagggaatagcaaactgtgactttgtatacagctatgacccgaatgtacttgagataa<br>tagagatagaaccgggagacataatagttgacccgaatcctgacaagagctttgatactgcagtatatcc<br>tgacagaaagataatagtattcctgtttgcagaagacagcggaacgggagcgtatgcaataactaaaga<br>cggagtatttgctacgatagtagcgaaagtaaaagaaggagcacctaacggactcagtgtaatcaaattt<br>gtagaagtaggcggatttgcgaacaatgaccttgtagaacagaagacacagttctttgacggtggagta

	aatgttggagatacaacagtacctacaacatcgccgacaacaacaccgccagagccgacgataactcc<br>gaacaagttgacacttaagataggcagagcagaaggaagacctggagacacggtggaaataccggtt<br>aacttgtatggagtacctcaaaaaggaatagcaagcggtgacttcgtagtaagctatgacccgaatgtac<br>ttgagataatagagatagaaccgggagaattgatagttgacccgaatcctaccaagagctttgatactgc<br>agtatatcctgacagaaagatgatagtattcctgtttgcggaagacagcggaacaggagcgtatgcaata<br>actgaagatggagtatttgctacgatagtagcgaaagtaaaagaaggagcacctgaaggattcagtgca<br>atagaaatttctgagtttggtgcatttgcagataatgatctggtagaagtggaaactgaccttatcaatggtg<br>gagtacttgta
3Ct	acagtcgagatcggcaaagttacagcagccgttggatcaaaagtagaaatacctataaccctgaaagg<br>agtgccatccaaaggaatggccaattgcgacttcgtattgggttatgatccaaatgtgctggaagtaaca<br>gaagtaaaaccaggaagcataataaaagatccggatcctagcaagagctttgatagcgcaatatatccg<br>gatcgaaagatgattgtatttctgtttgcagaagacagtggaagaggaacgtatgcaataactcaggatg<br>gagtatttgcaacaattgtagccactgtcaaatcagctgcagcggcaccgattactttgcttgaagtaggt<br>gcatttgcggacaacgatttagtagaaataagcacaacttttgtcgcgggcggagtaaatcttggtagttc<br>cgtaccgacaacacagccaaatgttccgtccgacggtgtggtagtagaaattggcaaagttacgggatc<br>tgttggaactacagttgaaatacctgtatatttcagaggagttccatccaaaggaatagcaaactgcgactt<br>tgtgttcagatatgatccgaatgtattggaaattatagggatagatcccggagacataatagttgacccga<br>atcctaccaagagctttgatactgcaatatatcctgacagaaagataatagtattcctgtttgcggaagaca<br>gcggaacaggagcgtatgcaataactaaagacggagtatttgcaaaaataagagcaactgtaaaatca<br>agtgctccgggctatattactttcgacgaagtaggtggatttgcagataatgacctggtagaacagaagg<br>tatcatttatagacggtggtgttaacgttggcaatgcaacaccgaccaagggagcaacaccaacaaata<br>cagctacgccgacaaaatcagctacggctacgcccaccaggccatcggtaccgacaaacacaccgac<br>aaacacaccggcaaatacaccggtatcaggcaatttgaaggttgaattctacaacagcaatccttcagat<br>actactaactcaatcaatcctcagttcaaggttactaataccggaagcagtgcaattgatttgtccaaactc<br>acattgagatattattatacagtagacggacagaaagatcagaccttctggtgtgaccatgctgcaataat<br>cggcagtaacggcagctacaacggaattacttcaaatgtaaaaggaacatttgtaaaaatgagttcctca<br>acaaataacgcagacacctaccttgaaataagctttacaggcggaactcttgaaccgggtgcacatgttc<br>agatacaaggtagatttgcaaagaatgactggagtaactatacacagtcaaatgactactcattcaagtct<br>gcttcacagtttgttgaatgggatcaggtaacagcatacttgaacggtgttcttgtatggggtaaagaacc<br>cggtggcagtgtagtaccatcaacacagcctgtaacaacaccacctgcaacaacaaaaccacctgcaa<br>caacaaaaccacctgcaacaacaataccgccgtcagatgatccgaatgcaataaagattaaggtggac<br>acagtaaatgcaaaaccgggagacacagtaaatatacctgtaagattcagtggtataccatccaaggga<br>atagcaaactgtgactttgtatacagctatgacccgaatgtacttgagataatagagataaaaccgggag<br>aattgatagttgacccgaatcctgacaagagctttgatactgcagtatatcctgacagaaagataatagtat<br>tcctgtttgcagaagacagcggaacaggagcgtatgcaataactaaagacggagtatttgctacgatagt<br>agcgaaagtaaaatccggagcacctaacggactcagtgtaatcaaatttgtagaagtaggcggatttgc<br>gaacaatgaccttgtagaacagaggacacagttctttgacggtggagtaaatgtt
4Ct	gtggtagtagaaattggcaaagttacgggatctgttggaactacagttgaaatacctgtatatttcagagg<br>agttccatccaaaggaatagcaaactgcgactttgtgttcagatatgatccgaatgtattggaaattatagg

	gatagatcccggagacataatagttgacccgaatcctaccaagagctttgatactgcaatatatcctgaca<br>gaaagataatagtattcctgtttgcggaagacagcggaacaggagcgtatgcaataactaaagacgga<br>gtatttgcaaaaataagagcaactgtaaaatcaagtgctccgggctatattactttcgacgaagtaggtgg<br>atttgcagataatgacctggtagaacagaaggtatcatttatagacggtggtgttaacgttggcaatgcaa<br>caccgaccaagggagcaacaccaacaaatacagctacgccgacaaaatcagctacggctacgccca<br>ccaggccatcggtaccgacaaacacaccgacaaacacaccggcaaatacaccggtatcaggcaattt<br>gaaggttgaattctacaacagcaatccttcagatactactaactcaatcaatcctcagttcaaggttactaat<br>accggaagcagtgcaattgatttgtccaaactcacattgagatattattatacagtagacggacagaaag<br>atcagaccttctggtgtgaccatgctgcaataatcggcagtaacggcagctacaacggaattacttcaaa<br>tgtaaaaggaacatttgtaaaaatgagttcctcaacaaataacgcagacacctaccttgaaataagcttta<br>caggcggaactcttgaaccgggtgcacatgttcagatacaaggtagatttgcaaagaatgactggagta<br>actatacacagtcaaatgactactcattcaagtctgcttcacagtttgttgaatgggatcaggtaacagcat<br>acttgaacggtgttcttgtatggggtaaagaacccggtggcagtgtagtaccatcaacacagcctgtaac<br>aacaccacctgcaacaacaaaaccacctgcaacaacaaaaccacctgcaacaacaataccgccgtca<br>gatgatccgaatgcaataaagattaaggtggacacagtaaatgcaaaaccgggagacacagtaaatat<br>acctgtaagattcagtggtataccatccaagggaatagcaaactgtgactttgtatacagctatgacccga<br>atgtacttgagataatagagataaaaccgggagaattgatagttgacccgaatcctgacaagagctttgat<br>actgcagtatatcctgacagaaagataatagtattcctgtttgcagaagacagcggaacaggagcgtatg<br>caataactaaagacggagtatttgctacgatagtagcgaaagtaaaatccggagcacctaacggactca<br>gtgtaatcaaatttgtagaagtaggcggatttgcgaacaatgaccttgtagaacagaggacacagttcttt<br>gacggtggagtaaatgttggagatacagctagcggagatacaacagaacctgcaacacctacaacacc<br>tgtaacaacaccgacaacaacagatgatctggatgcagtaaggattaaagtggacacagtaaatgcaaa<br>accgggagacacagtaagaatacctgtaagattcagcggtataccatccaagggaatagcaaactgtg<br>actttgtatacagctatgacccgaatgtacttgagataatagagatagaaccgggagacataatagttgac<br>ccgaatcctgacaagagctttgatactgcagtatatcctgacagaaagataatagtattcctgtttgcagaa<br>gacagcggaacgggagcgtatgcaataactaaagacggagtatttgctacgatagtagcgaaagtaaa<br>agaaggagcacctaacggactcagtgtaatcaaatttgtagaagtaggcggatttgcgaacaatgacctt<br>gtagaacagaagacacagttctttgacggtggagtaaatgttggagatacaacagtacctacaacatcg<br>ccgacaacaacaccgccagagccgacgataactccgaacaagttgacacttaagataggcagagcag<br>aaggaagacctggagacacggtggaaataccggttaacttgtatggagtacctcaaaaaggaatagca<br>agcggtgacttcgtagtaagctatgacccgaatgtacttgagataatagagatagaaccgggagaattg<br>atagttgacccgaatcctaccaagagctttgatactgcagtatatcctgacagaaagatgatagtattcctg<br>tttgcggaagacagcggaacaggagcgtatgcaataactgaagatggagtatttgctacgatagtagcg<br>aaagtaaaagaaggagcacctgaaggattcagtgcaatagaaatttctgagtttggtgcatttgcagata<br>atgatctggtagaagtggaaactgaccttatcaatggtggagtacttgta

Table S2. Scaffoldin genes sequences

Construction of plasmids for dockerin-containing enzymes expression. The gene encoding the dockerin domain of *Acetovibrio cellulolyticus* has been extracted using standard methods

and cloned following the gene encoding for *Zymomonas mobilis* alcohol dehydrogenase with a short 18 nucleotides linker yielding the zADH-Ac gene.

The gene encoding the dockerin domain module from Cel48S from *Clostridium thermocellum* has been extracted using standard methods and cloned following the gene encoding for *Pseudomonas putida* formaldehyde dehydrogenase with a short 33 nucleotides linker yielding the pFormDH-Ct gene.

The gene encoding the dockerin module from Cel48S from *Clostridium thermocellum* has been extracted using standard methods and cloned following the gene encoding for *E. coli* copper oxidase with a short 33 nucleotides linker yielding the CueO-Ct gene.

All the 3 enzymes genes were modified with the removal of the stop codon in order to enable the addition of a domain fused to the C-termini of the enzyme. *Nco*I and *Bam*HI restriction sites were added to genes and they were cloned to the pET15b expression vector yielding the pET15b-zADH-Ac, pET15b-pFromDH-Ct and pET15b-CueO-Ct vectors which have been transformed to BL21 strain *E. coli* bacteria for expression. All the genes with the modification described are listed in Table S3.

Gene	Sequence
zADH-Ac	atggcttcttcaactttttatattcctttcgtcaacgaaatgggcgaaggttcgcttgaaaaagcaatcaag<br>gatcttaacggcagcggctttaaaaatgcgctgatcgtttctgatgctttcatgaacaaatccggtgttgtg<br>aagcaggttgctgacctgttgaaagcacagggtattaattctgctgtttatgatggcgttatgccgaaccc<br>gactgttaccgcagttctggaaggccttaagatcctgaaggataacaattcagacttcgtcatctccctcg<br>gtggtggttctccccatgactgcgccaaagccatcgctctggtcgcaaccaatggtggtgaagtcaaag<br>actacgaaggtatcgacaaatctaagaaacctgccctgcctttgatgtcaatcaacacgacggctggtac<br>ggcttctgaaatgacgcgtttctgcatcatcactgatgaagtccgtcacgttaagatggccattgttgaccg<br>tcacgttaccccgatggtttccgtcaacgatcctctgttgatggttggtatgccaaaaggcctgaccgccg<br>ccaccggtatggatgctctgacccacgcatttgaagcttattcttcaacggcagctactccgatcaccgat<br>gcttgcgctttgaaagcagcttccatgatcgctaagaatctgaagaccgcttgcgacaacggtaaggata<br>tgccggctcgtgaagctatggcttatgcccaattcctcgctggtatggccttcaacaacgcttcgcttggtt<br>atgtccatgctatggctcaccagttgggcggttactacaacctgccgcatggtgtctgcaacgctgttctg<br>cttccgcatgttctggcttataacgcctctgtcgttgctggtcgtctgaaagacgttggtgttgctatgggtc<br>tcgatatcgccaatctcggtgataaagaaggcgcagaagccaccattcaggctgttcgcgatctggctg<br>cttccattggtattccagcaaacctgaccgagctgggtgctaagaaagaagatgtgccgcttcttgctga<br>ccacgctctgaaagatgcttgtgctctgaccaacccgcgtcagggtgatcagaaagaagttgaagaact<br>cttcctgagcgctttcggtaccaaatttatatatggtgatgttgatggtaatggaagtgtaagaattaatgat<br>gctgtcctaataagagactatgtattaggaaaaatcaatgaattcccatatgaatatggtatgcttgcagca

	gatgttgatggtaatggaagtataaaaattaatgatgctgttctagtaagagactacgtgttaggaaagata<br>tttttattccctgttgaagagaaagaagaataa
pFormDH-Ct	atggcgtcaggcaaccgtggcgtggtgtatctgggctctggcaaagtggaagttcaaaaaatcgactac<br>ccgaaaatgcaggacccgcgtggcaagaaaattgaacatggcgtgatcctgaaagtggtttcaaccaa<br>catttgtggctcggatcagcacatggttcgtggtcgcaccacggcacaagtgggtctggttctgggtcac<br>gaaattacgggcgaagttatcgaaaaaggtcgtgatgtcgaaaacctgcagatcggtgacctggtgag<br>cgttccgtttaatgtggcgtgcggccgttgtcgctcttgcaaagaaatgcataccggtgtgtgtctgacgg<br>ttaacccggctcgcgcgggcggtgcctatggctacgtcgatatgggtgactggaccggcggtcaagca<br>gaatatctgctggttccgtacgctgatttcaatctgctgaaactgccggatcgtgacaaagcgatggaaa<br>aaattcgcgatctgacctgcctgagtgacatcctgccgaccggttatcacggcgcagtcacggccggc<br>gtgggtccgggcagtacggtctacgtggcaggtgcaggtccggtgggtctggcggcggcggcatcc<br>gcccgtctgctgggcgcggccgtcgtgattgttggtgatctgaacccggcacgcctggctcatgcgaaa<br>gcccagggctttgaaattgctgatctgtcactggacaccccgctgcacgaacaaatcgcagctctgctg<br>ggtgaaccggaagtggattgcgcagtcgacgctgtgggcttcgaagcgcgtggtcatggccacgaag<br>gtgccaaacacgaagcaccggctaccgttctgaacagcctgatgcaggttacgcgcgtcgccggtaaa<br>attggcatcccgggtctgtatgtgaccgaagatccgggtgcagttgacgcggcggcaaaaattggtagc<br>ctgtctatccgttttggtctgggctgggcgaaaagtcattccttccacaccggccagacgccggtgatga<br>aatataaccgtgcgctgatgcaagccattatgtgggatcgcattaatatcgcagaagttgtcggtgttcag<br>gtcatctctctggatgacgcaccgcgcggttacggtgaatttgatgccggtgtcccgaaaaaattcgttat<br>tgacccgcacaaaaccttctcagccgccggtacccactgtactagtacatataaagtacctggtactcctt<br>ctactaaattatacggcgacgtcaatgatgacggaaaagttaactcaactgacgctgtagcattgaagag<br>atatgttttgagatcaggtataagcatcaacactgacaatgccgatttgaatgaagacggcagagttaatt<br>caactgacttaggaattttgaagagatatattctcaaagaaatagatacattgccgtacaagaactaa
CueO-Ct	atggaacgtcgtgatttcttaaaatattccgtcgcgctgggtgtggcttcggctttgccgctgtggagccg<br>cgcagtatttgcggcagaacgcccaacgttaccgatccctgatttgctcacgaccgatgcccgtaatcgc<br>attcagttaactattggcgcaggccagtccacctttggcgggaaaactgcaactacctggggctataacg<br>gcaatctgctggggccggcggtgaaattacagcgcggcaaagcggtaacggttgatatctacaaccaa<br>ctgacggaagagacaacgttgcactggcacgggctggaagtaccgggtgaagtcgacggcggcccg<br>cagggaattattccgccaggtggcaagcgctcggtgacgttgaacgttgatcaacctgccgctacctgc<br>tggttccatccgcatcagcacggcaaaaccgggcgacaggtggcgatggggctggctgggctggtgg<br>tgattgaagatgacgagatcctgaaattaatgctgccaaaacagtggggtatcgatgatgttccggtgatc<br>gttcaggataagaaatttagcgccgacgggcagattgattatcaactggatgtgatgaccgccgccgtg<br>ggctggtttggcgatacgttgctgaccaacggtgcaatctacccgcaacacgctgccccgcgtggttgg<br>ctgcgcctgcgtttgctcaatggctgtaatgcccgttcgctcaatttcgccaccagcgacaatcgcccgct<br>gtatgtgattgccagcgacggtggtctgctacctgaaccagtgaaggtgagcgaactgccggtgctgat<br>gggcgagcgttttgaagtgctggtggaggttaacgataacaaaccctttgacctggtgacgctgccggt<br>cagccagatggggatggcgattgcgccgtttgataagcctcatccggtaatgcggattcagccgattgct<br>attagtgcctccggtgctttgccagacacattaagtagcctgcctgcgttaccttcgctggaagggctgac

	ggtacgcaagctgcaactctctatggacccgatgctcgatatgatggggatgcagatgctaatggagaa<br>atatggcgatcaggcgatggccgggatggatcacagccagatgatgggccatatggggcacggcaat<br>atgaatcatatgaaccacggcgggaagttcgatttccaccatgccaacaaaatcaacggtcaggcgtttg<br>atatgaacaagccgatgtttgcggcggcgaaagggcaatacgaacgttgggttatctctggcgtgggc<br>gacatgatgctgcatccgttccatatccacggcacgcagttccgtatcttgtcagaaaatggcaaaccgc<br>cagcggctcatcgcgcgggctggaaagataccgttaaggtagaaggtaatgtcagcgaagtgctggtg<br>aagtttaatcacgatgcaccgaaagaacatgcttatatggcgcactgccatctgctggagcatgaagata<br>cggggatgatgttagggtttacggtaggtaccactagtacatataaagtacctggtactccttctactaaat<br>tatacggcgacgtcaatgatgacggaaaagttaactcaactgacgctgtagcattgaagagatatgttttg<br>agatcaggtataagcatcaacactgacaatgccgatttgaatgaagacggcagagttaattcaactgact<br>taggaattttgaagagatatattctcaaagaaatagatacattgccgtacaagaactaa

Table S3. Dockerin-containing genes sequences

Yeast transformation. Yeast transformation was performed using the lithium acetate method. A freshly streaked EBY100 colony was inoculated in 5mL YPD media and grown overnight at 30°C. The overnight culture was used to inoculate 100mL YPD media to OD_{600} of 0.1 after which the cells were grown at 30°C to OD_{600} of 1.2. Cells were harvested by centrifugation at 4000rpm for 5min at 4°C, resuspended in 50mL H_2O, centrifuged again, resuspended in 1mL H_2O, harvested (2min, max speed) and resuspended in 300µL H_2O. Each transformation reaction contained 100µL treated yeast cells, 480µL PEG 50%, 72µL LiAc 1M, 10µL 10mg/mL herring sperm DNA, 30µL H_2O, 30µL plasmid (200-300ng). After a short vortex, the reaction was incubated for 30 min at 30°C. 30µL DMSO were added and the reaction was incubated for 15min at 42°C with continuous shaking. After a 30 s spin at max speed, the pellet was resuspended in 200µL H_2O and the cells plated onto selective SC ura^- trp^- plates and grown at 30°C for 48hr.

Yeast surface display induction and Fluorescence Activated Cell Sorting (FACS). Transformed yeast were grown overnight in 5.0 mL SDCAA at 30°C. 1.0 mL of culture was harvested by centrifugation and resuspended in 5mL of induction media. Induced culture was grown with shaking (250 rpm) for at least 18 hrs, at different temperatures: 20°C, 30°C, 37°C.

1.0 mL of induced yeast cells, which absorbance at a wavelength of 600nm has been approximately 1.0, was collected by centrifugation. The pellet was washed with 0.5 mL PBSF (rinsed, and pelleted again). The yeasts were labeled with mouse anti-c-Myc IgG1 in 50µL of PBSF. Re-suspended cells were incubated for 1hr at 25°C and 400rpm. Yeast cells were collected by centrifugation and washed twice with ice cold PBSF. After addition of secondary reagents (goat anti-mouse PE conjugate), cells were incubated on ice and protected from light

exposure for 1hr. After two washes with ice cold PBSF, the cells were resuspended in 0.5mL ice-cold PBSF. A 70μL cell suspension was diluted in 0.5mL PBS and the mixture analyzed by an 'Eclipse' flow cytometer (iCyt, IL, USA).

Control cultures: the positive control for myc-labeled surface expressed proteins was yeast expressing glucoamilase (PCGA) on their surface (induced at 30°C). Negative controls were EBY100 yeast (grown in YPD, at 30°C) and induced yeast containing the different scaffoldins vectors incubated only with secondary antibody.

Results

FACS analysis. The surface expression in the YSD system can be measured through immunofluorescence labeling of either the c-myc epitope or the hemagglutinin tag flanking the expressed protein. The c-myc epitope was marked using mouse anti c-myc antibody to which PE labeled goat anti mouse antibody has been bound. Figure S1 shows the flow cytometry histograms of the assays performed with different yeast displaying the pCTL20(-CBM) (Fig. S1-A), pCT2Ct (Fig. S1-B), pCT3Ct (Fig. S1-C) and pCT4Ct (Fig. S1-D), encoding for chimeric scaffoldins and di-, tri- and tetravalent mini-scaffoldins, respectively. In all assays the induction of the YSD system has been performed at 30°C. It can be observed that the surface display levels are two orders of magnitude higher than the negative controls of yeast not displaying on their surface. Background fluorescence of the cells has been tested using scaffoldin displaying yeast that were not incubated with the secondary antibody and they were found to have fluorescence in levels similar to the negative control of yeast without YSD.

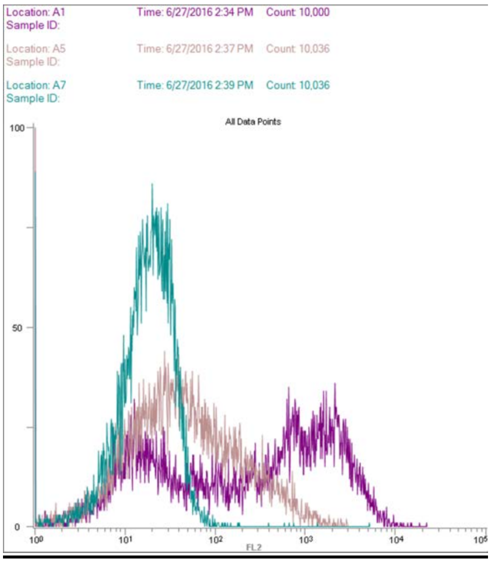

Figure S1-A. Histogram analysis of c-myc-tag labeled EBY100 cells: without added plasmid (negative control, cyan), cells displaying PCNA (positive control, pink-gray), cells displaying the L20(-CBM) chimeric scaffoldin (purple).

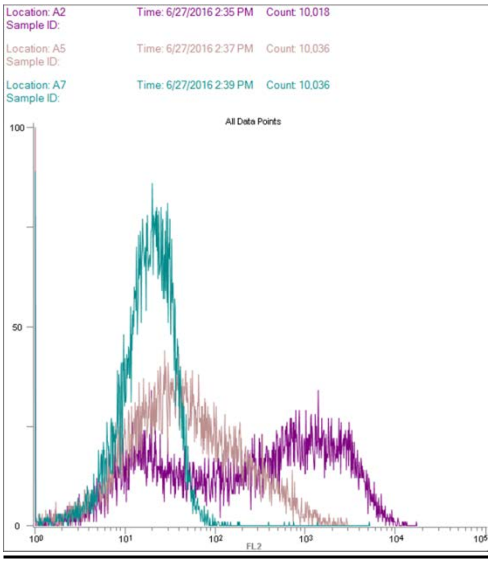

Figure S1-B. Histogram analysis of c-myc-tag labeled EBY100 cells: without added plasmid (negative control, cyan), cells displaying PCNA (positive control, pink-gray), cells displaying the divalent mini-scaffoldin (purple).

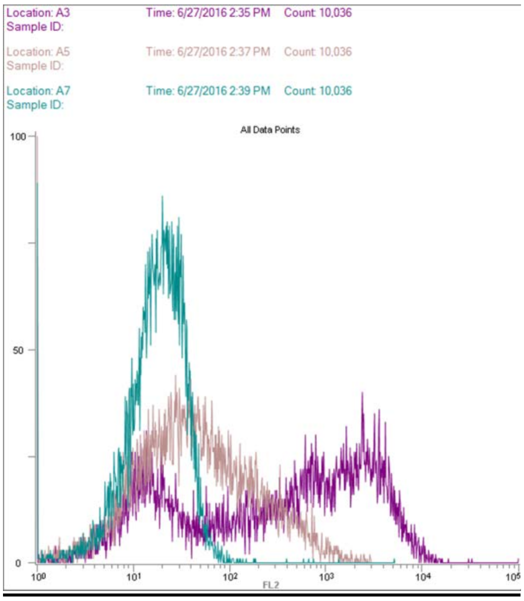

Figure S1-C. Histogram analysis of c-myc-tag labeled EBY100 cells: without added plasmid (negative control, cyan), cells displaying PCNA (positive control, pink-gray), cells displaying the trivalent mini-scaffoldin (purple).

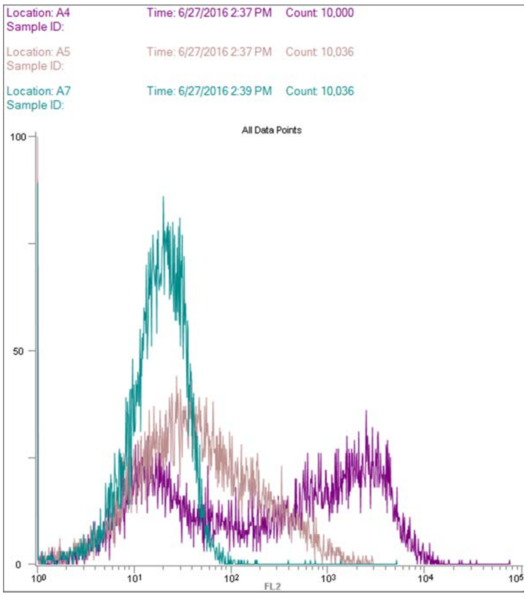

Figure S1-D. Histogram analysis of c-myc-tag labeled EBY100 cells: without added plasmid (negative control, cyan), cells displaying PCNA (positive control, pink-gray), cells displaying the tetravalent mini-scaffoldin (purple).

Biochemical activity of dockerin-containing zADH and pFormDH in bacteria lysate. The activity of both enzymes towards their substrates has been validated using a colorimetric assay as described in the manuscript, following the change in absorbance at 340nm due to NAD^+ reduction. Figure S2 shows the activity of zADH-Ac towards ethanol (Fig. S2-A) and pFormDH-Ct towards formaldehyde (Fig S2-B). In all the assays the enzymes have shown activity comparing to a negative control of a lysate of bacteria not expressing the enzymes. The dockerin modification has resulted in an activity loss compare to the wild-type enzyme of 33% for the zADH (Fig. S2-A (i,ii)) and 75% for the pFormDH (Fig. S2-B).

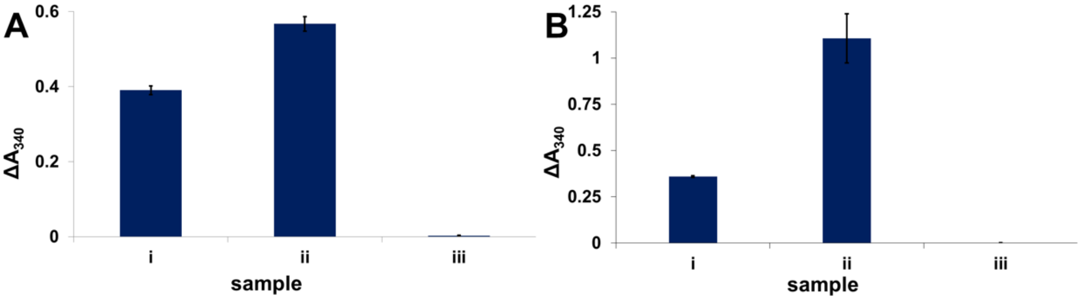

Figure S2. Changes in absorbance at 340nm following the reduction of 2.1mM NAD^+ reduction as a result of (A) 1% v/v EtOH oxidation in the presence of (i) zADH-Ac expressing; (ii) Wild-type zADH expressing or (iii) native bacterial lysates; (B) 0.002% v/v formaldehyde oxidation in the presence of (i) pFormDH-Ct expressing; (ii) wild-type pFormDH expressing or (iii) native bacterial lysate. Assay performed at 30°C in 50mM Tris pH 8 with 10 mM $CaCl_2$.

After the activity of the pFormDH-Ct has been demonstrated towards its preferable substrate formaldehyde, the activity towards acetaldehyde which is required for the cascade was validated using a similar assay. Figure S3 shows the activity of the dockerin-containing enzyme (Fig. S3 (i)) comparing to the activity in a wild-type bacterial lysate (Fig. S3 (ii)).

Cross-reactivity of the enzymes: zADH-Ac towards aldehydes or pFormDH-Ct towards EtOH was not observed in any of the assays.

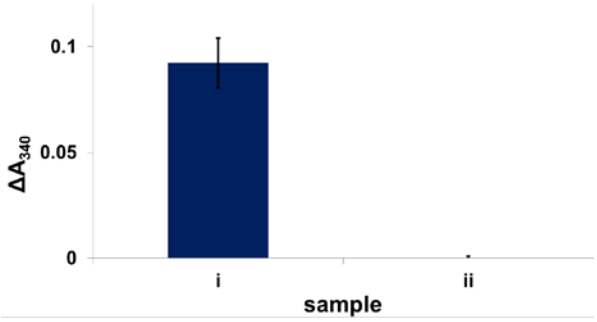

Figure S3. Changes in absorbance at 340nm following the reduction of 2.1mM NAD^+ reduction as a result of acetaldehyde 50mM oxidation in the presence of (i) pFormDH-Ct expressing or (ii) native bacterial lysate. Assay performed at 30°C in 50mM Tris pH 8 with 10mM $CaCl_2$.

Biochemical activity of dockerin-containing CueO in bacteria lysate. The activity of the dockerin-containing CueO-Ct was validated using a colorimetric assay as described in the manuscript, following the change in absorbance at 430nm due to OPD oxidation. Figure S4 shows an increase in the absorbance as a result of OPD oxidation in the presence of CueO-Ct expressing bacteria lysate (Fig. S4 (i)). This activity was a little lower than the activity of the native enzyme (Fig. S4 (ii)) and reflects approx. 20% loss. As a negative control a lysate of wild-type bacterial lysate was used and a much lower activity was observed (Fig. S4 (iii)) which is a result of the basal expression of the wild-type enzyme in *E. coli*.

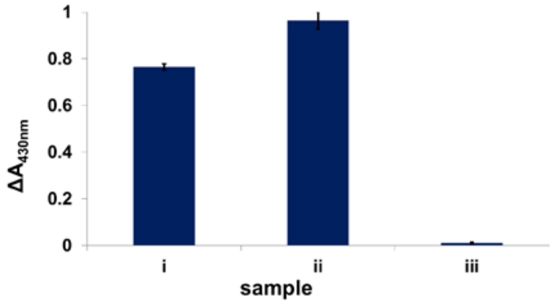

Figure S4. Changes in absorbance at 430nm following 3.7mM OPD oxidation in the presence of oxygen and (i) CueO-Ct expressing; (ii) wild-type CueO expressing or (iii) native bacterial lysate. Assay performed at 30°C in acetate 100mM $CuSO_4$ 0.4mM pH 5.

Power output vs. current density curves of the EtOH oxidation cascade cells.

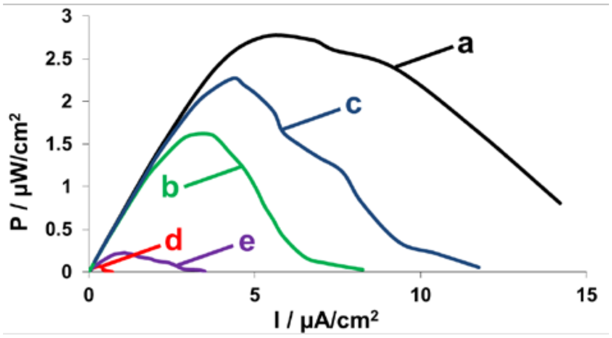

Figure S5. Performance of yeast surface-displayed enzyme cascade. Power output vs. current density curves of anodes of scaffoldin-displaying yeast that were incubated with (a) a mixture of zADH-Ac and pFormDH-Ct expressing bacterial lysates; (b) zADH-Ac expressing bacterial lysate alone; (c) pFormDH-Ct expressing bacterial lysate alone; (d) native bacterial lysate; (e) native yeast incubated with both zADH-Ac and pFormDH-Ct expressing bacteria lysates. EtOH 2% v/v as fuel, NAD^+ 1.05 mM and 1 mM MB have been used as the enzymes cofactor and redox mediator respectively.

Power output vs. current density curves of the CueO multiple copies displaying yeast.

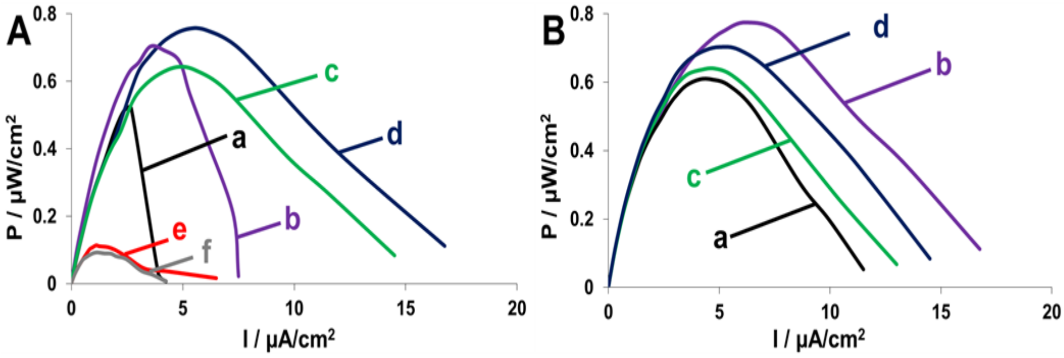

Figure S6. Performance of biocathodes comprising yeast cells containing increasing copies of surface-displayed CueO. Power output vs. current density curves for (A) different scaffoldin-displaying yeast with (a) mono-valent; (b) bi-valent; (c) tri-valent; and (d) tetra-valent scaffoldins with CueO-Ct; (e) native yeast with CueO-Ct; (f) mono-valent scaffoldin-bearing yeast incubated with native CueO. (B) Power output vs. current density curves of cells with antimycin A and with (a) mono-valent; (b) di-valent; (c) tri-valent; (d) tetra-valent mini-scaffoldins. Air purged to the cells, Cu^{2+} 25 μM as redox mediator and enzyme cofactor.

References:

1. Boder, T. E.; Wittrup, D. K. Yeast surface display for screening combinatorial polypeptide libraries. *Nat. Biotechnol.* **1997**, *15*, 553–557.

2. Bahartan, K.; Amir, L.; Israel, A.; Lichtenstein, R. G.; Alfonta, L. In Situ fuel processing in a microbial fuel cell. *ChemSusChem* **2012**, *5*, 1820–1825.

3. Amir, L.; Carnally, S. a.; Rayo, J.; Rosenne, S.; Melamed Yerushalmi, S.; Schlesinger, O.; Meijler, M. M.; Alfonta, L. Surface display of a redox enzyme and its site-specific wiring to gold electrodes. *J. Am. Chem. Soc.* **2013**, *135*, 70–73.

4. Sherman, F. Getting started with yeast. *Methods Enzymol.* **2002**, *350*, 3–41.

5. Vazana, Y.; Barak, Y.; Unger, T.; Peleg, Y.; Shamshoum, M.; Ben-Yehezkel, T.; Mazor, Y.; Shapiro, E.; Lamed, R.; Bayer, E. A. A synthetic biology approach for evaluating the functional contribution of designer cellulosome components to deconstruction of cellulosic substrates. *Biotechnol. Biofuels* **2013**, *6*, 1–18.
